# Transcriptional Profiles of a Foliar Fungal Endophyte (*Pestalotiopsis*, Ascomycota) and Its Bacterial Symbiont (*Luteibacter*, *Gammaproteobacteria*) Reveal Sulfur Exchange and Growth Regulation during Early Phases of Symbiotic Interaction

**DOI:** 10.1128/msystems.00091-22

**Published:** 2022-03-16

**Authors:** Justin P. Shaffer, Morgan E. Carter, Joseph E. Spraker, Meara Clark, Brian A. Smith, Kevin L. Hockett, David A. Baltrus, A. Elizabeth Arnold

**Affiliations:** a School of Plant Sciences, The University of Arizona, Tucson, Arizona, USA; b Department of Pediatrics, School of Medicine, University of California, La Jolla, California, USA; c Hexagon Bio, Menlo Park, California, USA; d Department of Plant Pathology and Environmental Microbiology, Pennsylvania State University, Pennsylvania, USA; e Department of Ecology and Evolutionary Biology, The University of Arizona, Tucson, Arizona, USA; Purdue University

**Keywords:** bacterial-fungal interactions, culture conditions, endobacteria, gene expression, interdomain, interkingdom, *Platycladus orientalis*, RNA-seq, symbiosis, transcriptomics

## Abstract

Symbiosis with bacteria is widespread among eukaryotes, including fungi. Bacteria that live within fungal mycelia (endohyphal bacteria) occur in many plant-associated fungi, including diverse Mucoromycota and Dikarya. *Pestalotiopsis* sp. strain 9143 is a filamentous ascomycete isolated originally as a foliar endophyte of Platycladus orientalis (Cupressaceae). It is infected naturally with the endohyphal bacterium *Luteibacter* sp. strain 9143, which influences auxin and enzyme production by its fungal host. Previous studies have used transcriptomics to examine similar symbioses between endohyphal bacteria and root-associated fungi such as arbuscular mycorrhizal fungi and plant pathogens. However, currently there are no gene expression studies of endohyphal bacteria of Ascomycota, the most species-rich fungal phylum. To begin to understand such symbioses, we developed methods for assessing gene expression by *Pestalotiopsis* sp. and *Luteibacter* sp. when grown in coculture and when each was grown axenically. Our assays showed that the density of *Luteibacter* sp. in coculture was greater than in axenic culture, but the opposite was true for *Pestalotiopsis* sp. Dual-transcriptome sequencing (RNA-seq) data demonstrate that growing in coculture modulates developmental and metabolic processes in both the fungus and bacterium, potentially through changes in the balance of organic sulfur via methionine acquisition. Our analyses also suggest an unexpected, potential role of the bacterial type VI secretion system in symbiosis establishment, expanding current understanding of the scope and dynamics of fungal-bacterial symbioses.

**IMPORTANCE** Interactions between microbes and their hosts have important outcomes for host and environmental health. Foliar fungal endophytes that infect healthy plants can harbor facultative endosymbionts called endohyphal bacteria, which can influence the outcome of plant-fungus interactions. These bacterial-fungal interactions can be influential but are poorly understood, particularly from a transcriptome perspective. Here, we report on a comparative, dual-RNA-seq study examining the gene expression patterns of a foliar fungal endophyte and a facultative endohyphal bacterium when cultured together versus separately. Our findings support a role for the fungus in providing organic sulfur to the bacterium, potentially through methionine acquisition, and the potential involvement of a bacterial type VI secretion system in symbiosis establishment. This work adds to the growing body of literature characterizing endohyphal bacterial-fungal interactions, with a focus on a model facultative bacterial-fungal symbiosis in two species-rich lineages, the Ascomycota and *Proteobacteria*.

## INTRODUCTION

Symbioses between eukaryotes and bacteria are widespread, with profound impacts ranging from the benefits of the gut microbiome with respect to human health to the cost of plant pathogens to global agriculture (1, 2). The molecular mechanisms underlying relationships ranging from antagonism to mutualism have been studied for decades in animals and plants, including the ways in which pathogenic and beneficial microbes establish in a new host. Although ubiquitous in nature, bacterial-fungal interactions remain relatively poorly understood, despite growing knowledge of their contributions to the emergent properties of microbiomes (3, 4). For example, bacteria living with fungi inhabiting plant roots and leaves can influence fungal phenotypes, including growth, reproduction, and pathogenicity, as well as the outcomes of plant-fungus interactions (5–9).

Despite “bacterium-like organelles” being discovered in arbuscular mycorrhizal fungi (AMF) decades ago (10), only recently have endohyphal bacteria (EHB) been identified living intracellularly in diverse plant-associated fungi. To date, members of the Mucoromycota, Basidiomycota, and Ascomycota have been identified as hosts to EHB, including *Proteobacteria*, *Firmicutes*, *Tenericutes*, *Bacteroidetes*, and others (11–15). Thus, both the capacity of bacteria to live within fungal hyphae and the capacity of diverse fungi to harbor bacterial endosymbionts are phylogenetically widespread and functionally diverse (3, 16). For example, one EHB associated with AMF, “*Candidatus* Glomeribacter gigasporarum” (*Betaproteobacteria*), is a vertically transmitted, obligate biotroph with a reduced genome (5, 11). In contrast, diverse EHB cultured from or observed in ectomycorrhizal fungi and foliar fungal endophytes appear to be facultatively associated with fungal hosts, with relatively unreduced genomes (13, 17, 18). Even among these facultative interactions, the impacts on fungal hosts by EHB include alterations in carbon use, growth of germinating spores, degradation of plant cell wall compounds, and sporulation (7, 8, 19, 20). The metabolic, proteomic, and transcriptomic changes that facilitate these associations and the associated bacterial and fungal phenotypes are not well known. Changes to fungal traits by the presence of a bacterial symbiont may impact other organisms, such as a plant host through increased virulence (21) or plant growth promotion (22, 23). Therefore, understanding these bacterial-fungal interactions will expand knowledge of fungal ecology more broadly.

*Mycetohabitans* spp. (formerly *Burkholderia*, *Betaproteobacteria*) and their host, Rhizopus microsporus (Mucoromycotina), represent one emerging model system for EHB based on the ability to independently culture and reintroduce the partners *in vitro* (3). Metabolic and transcriptomic studies have revealed changes in fungal lipid metabolism underlying their partnership formation, and a lack of reactive oxygen species burst in compatible partners (24, 25). The unique requirement of *Mycetohabitans* spp. for R. microsporus sporulation provides a context for probing genes involved in fungal reproduction in a genetically recalcitrant fungal clade (26). Essential bacterial genes for symbiosis establishment have been identified in *Mycetohabitans* spp., namely, type II and type III secretion systems (T2SS and T3SS, respectively) required for invasion of fungal hyphae (27, 28). The T2SS secretes chitinases critical for bacterial entry, but only one T3SS effector protein has been characterized, and it is not required for the establishment of symbiosis (28, 29). Yet, many EHB outside the *Burkholderiales* lack either or both T2SS and T3SS (3, 18). Indeed, the phylogenetic diversity of both EHB and their fungal hosts suggests bacteria can use many yet undiscovered methods to establish in a given fungus, with a variety of potential outcomes. For example, a lipopeptide produced by Ralstonia solanacearum, ralsolamycin, induces chlamydospore formation in and facilitates invasion of multiple fungi (30).

An additional emerging model for EHB is *Luteibacter* sp. strain 9143 (*Xanthomonadaceae*, *Gammaproteobacteria*) and its host, the foliar fungal endophyte *Pestalotiopsis* sp. strain 9143 (Sporocadaceae, Xylariales, Ascomycota) (13). The *Luteibacter*-*Pestalotiopsis* interaction represents the typical facultative, horizontally transmitted life modes of EHB found in diverse Dikarya (3). Unlike *Mycetohabitans* spp., *Luteibacter* sp. strain 9143 does not have a T3SS, but it does have type I, II, IV, and VI secretion systems (18). *Luteibacter* sp. strain 9143 has not been observed to be vertically transmitted in its host (i.e., it is not observed readily in conidia). It can be isolated reliably in culture (31). *Luteibacter* sp. strain 9143 increases the ability of *Pestalotiopsis* sp. strain 9143 to establish as an endophyte in plant hosts (3) and enhances its production of indole-3-acetic acid (22). In addition, *Pestalotiopsis* sp. strain 9143 harboring *Luteibacter* sp. strain 9143 exhibits increased cellulase activity, growth on cellulose-enriched growth medium, and degradation of senescent leaf tissue (3).

In this study, we contextualized phenotypic observations by using a transcriptomic approach to consider gene expression of *Pestalotiopsis* sp. strain 9143 and its facultative EHB, *Luteibacter* sp. strain 9143, when grown axenically and in coculture. Here, the coculture condition represents an *in vitro* account of the early stages of the interaction, as it includes free-living, externally associated, and endohyphal cells of *Luteibacter* sp. strain 9143, as well as cells of *Pestalotiopsis* sp. strain 9143 responding to those different bacterial phases. We used transcripts from axenic cultures of the fungus to optimize assembly and annotation of its genome and used that modified assembly to map fungal transcripts in parallel to mapping bacterial transcripts to a previously published genome sequence for *Luteibacter* sp. strain 9143 (18). Our study was designed to test three main predictions. In previous phenotypic assays, we observed that *Luteibacter* sp. strain 9143 emerges from its fungal host under conditions of stress and grows readily in a free-living state, provided there is a sulfur source besides inorganic sulfate (19, 31, 32). Therefore, we predicted that the bacterium would be mildly parasitic on the fungal partner, as illustrated by enhanced bacterial growth but reduced fungal growth in coculture relative to the axenic state. In such a situation, we would expect upregulation of genes associated with bacterial growth in coculture and changes in metabolic genes of both partners related to molecular exchange of sulfur compounds or other metabolites. Second, we predicted that genes upregulated in coculture would reflect symbiosis-relevant genes, especially if clustered or relevant to secretion systems or nutrient processing and coinciding with downregulation of genes associated with motility. In the fungus, we would expect upregulation of cellular repair mechanisms or other responses to infection and changes to secondary metabolite genes relevant to symbiotic establishment, such as upregulation of putative signaling molecules. Third, we predicted transcriptional changes related to carbohydrates such as cellulose, based on observed differences in cellulase activity of *Pestalotiopsis* sp. strain 9143 with and without *Luteibacter* (3). To address these predictions, we performed transcriptome sequencing (RNA-seq) and analysis of differential gene expression comparing *Luteibacter* sp. strain 9143 and *Pestalotiopsis* sp. strain 9143 grown together in cococulture versus separately (axenically). Here, we report on the genome of *Pestalotiopsis* sp. strain 9143 as well as the results of our analysis of differential gene expression.

## RESULTS

### Report of the genome of *Pestalotiopsis* sp. strain 9143.

We generated and assembled the genome sequence of *Pestalotiopsis* sp. strain 9143 grown axenically and following antibiotic treatment (GenBank accession no. JAHZSN000000000.1) by hybrid assembly of Illumina and Oxford Nanopore reads (see Materials and Methods). The final genome assembly was 46.3 Mbp, with 13,076 predicted protein coding genes and 247 tRNAs (Table 1). The BUSCO score, an assessment of genome completeness, was 94.6%, representing 1,255 complete genes out of 1,312 (see Table S1 in the supplemental material) in the Dikarya subkingdom data set (33). Profiling of secondary metabolite clusters using antiSMASH (34) revealed 64 secondary metabolite regions, 4 of which included neighboring protoclusters (see Table S2 in the supplemental material).

**TABLE 1:** Final statistics for the genome of *Pestalotiopsis* sp. strain 9143

Parameter	Result for parameter
Genome assembly	
Unique scaffolds, no.	104
Final assembly size, bp	46,255,514
Masked repeats, bp (%)	1,379,150 bp (2.98)
*N*_50_	817,980
GC content, %	52

Gene models, no.	
Protein coding	13,076 chromosomal, 40 mitochondrial
tRNAs	201 chromosomal, 65 mitochondrial

10.1128/msystems.00091-22.3 TABLE S1 BUSCO metrics for the genome of *Pestalotiopsis* sp. strain 9143 based on the dikarya_odb9 data set. Download Table S1, XLSX file, 0.01 MB. Copyright © 2022 Shaffer et al. 2022 Shaffer et al. https://creativecommons.org/licenses/by/4.0/ This content is distributed under the terms of the Creative Commons Attribution 4.0 International license.

10.1128/msystems.00091-22.4 TABLE S2 Summary of secondary metabolite clusters in the *Pestalotiopsis* sp. strain 9143 genome, predicted by antiSMASH. Download Table S2, XLSX file, 0.01 MB. Copyright © 2022 Shaffer et al. 2022 Shaffer et al. https://creativecommons.org/licenses/by/4.0/ This content is distributed under the terms of the Creative Commons Attribution 4.0 International license.

### Bacterial density and total biomass of experimental cultures supports mild parasitism of *Luteibacter* on *Pestalotiopsis*.

**FIG 1:**
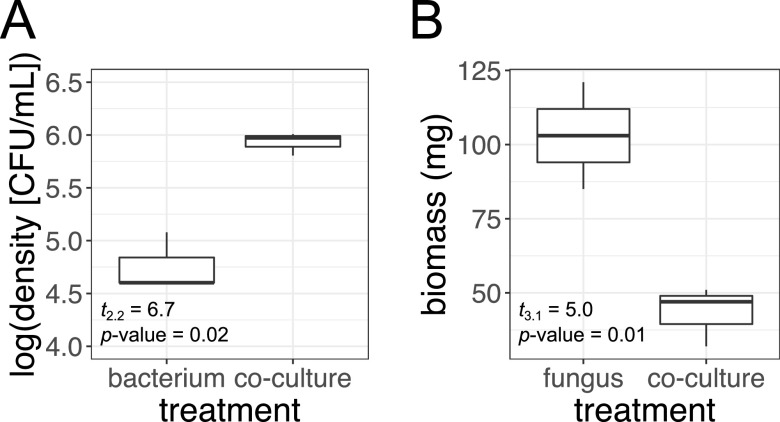
Differences in microbial density/biomass during coculture versus axenic growth. (A) Density (CFU/mL) of free-living *Luteibacter* sp. strain 9143 (i.e., cells not associated with fungal mycelium) when grown axenically (bacterium) versus with *Pestalotiopsis* sp. strain 9143 (coculture). (B) Biomass (mg) of *Pestalotiopsis* sp. strain 9143 when grown axenically (fungus) versus with *Luteibacter* sp. strain 9143 (coculture). The same volume of liquid growth medium was initially added to each sample. Biomass represents dry mass obtained by filtering cultures to remove the growth medium and lyophilizing the fresh biomass that was retained.

Prior to extracting RNA for analysis of differential gene expression, we examined the growth of *Luteibacter* sp. strain 9143 and *Pestalotiopsis* sp. strain 9143 when grown together in cococulture as well as when each was grown axenically. The bacterium was not observed in the axenic fungal culture, nor was the fungus present in the axenic bacterial culture. The average density of free-living *Luteibacter* sp. strain 9143 (i.e., cells not associated with fungal mycelium) in coculture flasks was significantly greater than that in axenic culture flasks (Welch’s *t* test, *t* = 6.7, *df* = 2.2, and *P* = 0.02) (Fig. 1A). The average density of free-living *Luteibacter* sp. strain 9143 in coculture flasks was 8.67 × 10^5^ ± 2.00 × 10^5^ CFU/mL, and that of *Luteibacter* sp. strain 9143 in axenic culture flasks was 6.67 × 10^4^ ± 4.62 × 10^4^ CFU/mL. The average dried biomass of *Luteibacter* sp. strain 9143 in axenic bacterial culture flasks was 3.3 ± 0.0 mg. In contrast, we observed significantly reduced growth of *Pestalotiopsis* sp. strain 9143 in coculture relative to axenic culture. The average combined biomass of *Pestalotiopsis* sp. strain 9143 and *Luteibacter* sp. strain 9143 in coculture flasks was 43.3 ± 0.01 mg, significantly less than the 103.00 ± 0.02 mg of *Pestalotiopsis* sp. strain 9143 in axenic culture flasks (Welch’s *t* test, *t* = 5.0, *df* = 3.1, and *P* = 0.01) (Fig. 1B).

### Transcriptional changes in *Luteibacter* sp. strain 9143 during coculture reflect increased growth and a symbiotic response.

**FIG 2:**
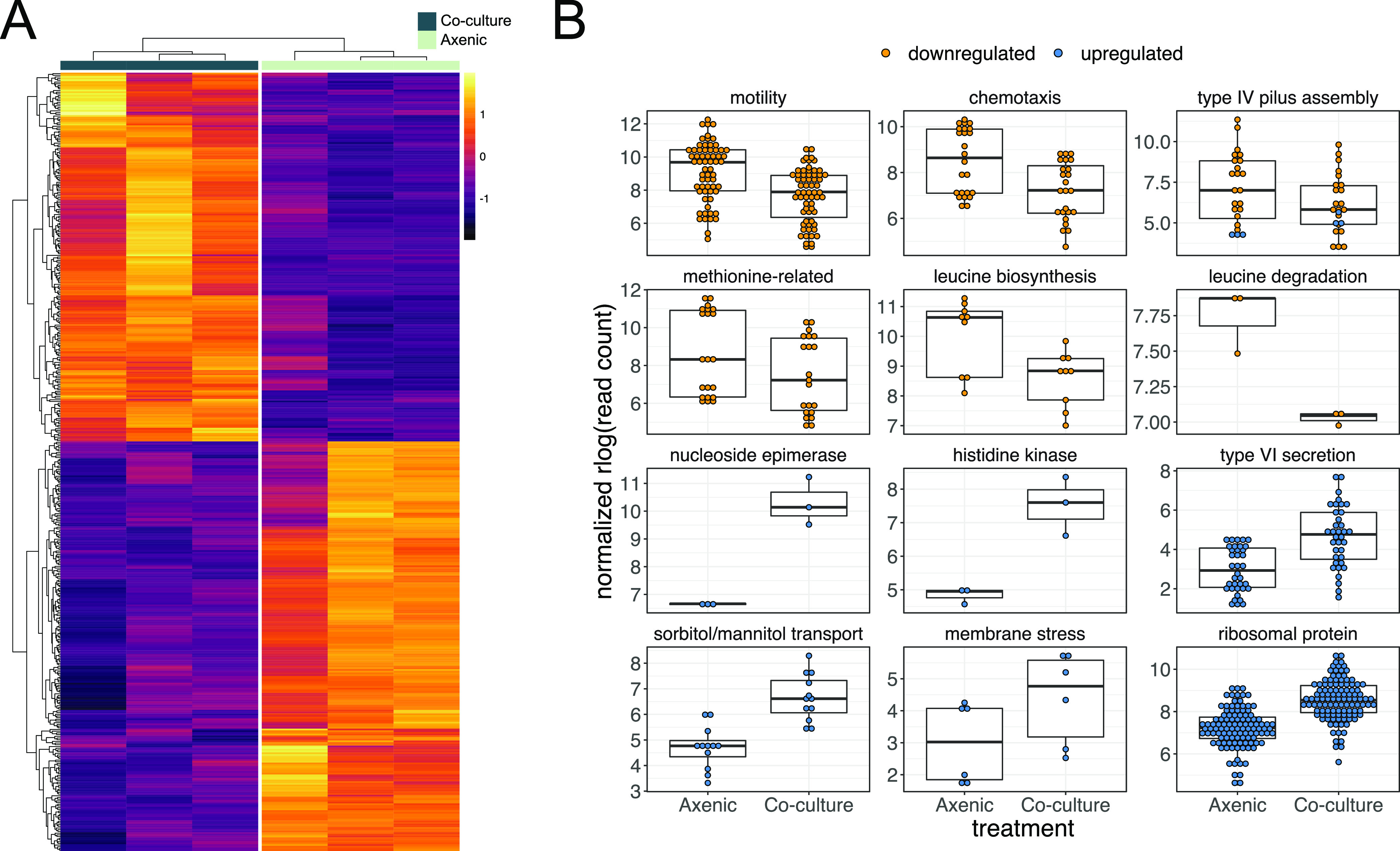
Summary of differentially expressed genes in *Luteibacter* sp. strain 9143 in coculture with *Pestalotiopsis* sp. strain 9143 compared to axenic growth. (A) Heat map showing the log fold change (i.e., DESeq2 regularized log [rlog] normalized) for each gene (rows) for each biological replicate (columns). (B) Normalized read counts for axenic versus cocultured *Luteibacter* sp. strain 9143 for specific groups of genes. Methionine-related genes include those for methionine biosynthesis, acquisition, and conversion. Each point represents the read count for a given gene from one replicate culture of a given treatment of *Luteibacter* sp. strain 9143 (i.e., axenic versus coculture): thus, three points are present for each gene.

During coculture with *Pestalotiopsis* sp. strain 9143, *Luteibacter* sp. strain 9143 upregulated 217 genes and downregulated 241 genes relative to its gene expression in the axenic state (i.e., for an adjusted *P* value or false-discovery rate [FDR] of 0.01) (Fig. 2; see Table S3 in the supplemental material). Our analysis of enrichment of Gene Ontology (GO) terms was largely uninformative, as nearly all terms identified as upregulated were also identified as downregulated (see Fig. S1 in the supplemental material). We observed that 37 of 54 (69%) genes predicted to code for ribosomal proteins were upregulated, with a log_2_ fold change (L2FC) of ∼1.5 to 3.0 (Table S3), which we speculate reflects enhanced bacterial growth in coculture (Fig. 1A). Twenty-eight of the 37 (76%) upregulated ribosomal protein genes are contained in a cluster with 4 additional ribosomal protein genes not differentially expressed (DE), whereas the remaining 9 are scattered throughout the genome (Table S3).

10.1128/msystems.00091-22.1 FIG S1 Results of Gene Ontology (GO) term enrichment analysis for *Luteibacter* sp. strain 9143. (A) Upregulated GO terms. (B) Downregulated GO terms. CC, cellular compartment; MF, molecular function. Download FIG S1, EPS file, 0.4 MB. Copyright © 2022 Shaffer et al. 2022 Shaffer et al. https://creativecommons.org/licenses/by/4.0/ This content is distributed under the terms of the Creative Commons Attribution 4.0 International license.

10.1128/msystems.00091-22.5 TABLE S3 Summary of differentially expressed genes in *Luteibacter* sp. strain 9143 when grown in coculture with *Pestalotiopsis* sp. strain 9143. Download Table S3, XLSX file, 0.5 MB. Copyright © 2022 Shaffer et al. 2022 Shaffer et al. https://creativecommons.org/licenses/by/4.0/ This content is distributed under the terms of the Creative Commons Attribution 4.0 International license.

### *Luteibacter* upregulates type VI secretion, signaling, and transport in coculture.

**FIG 3:**
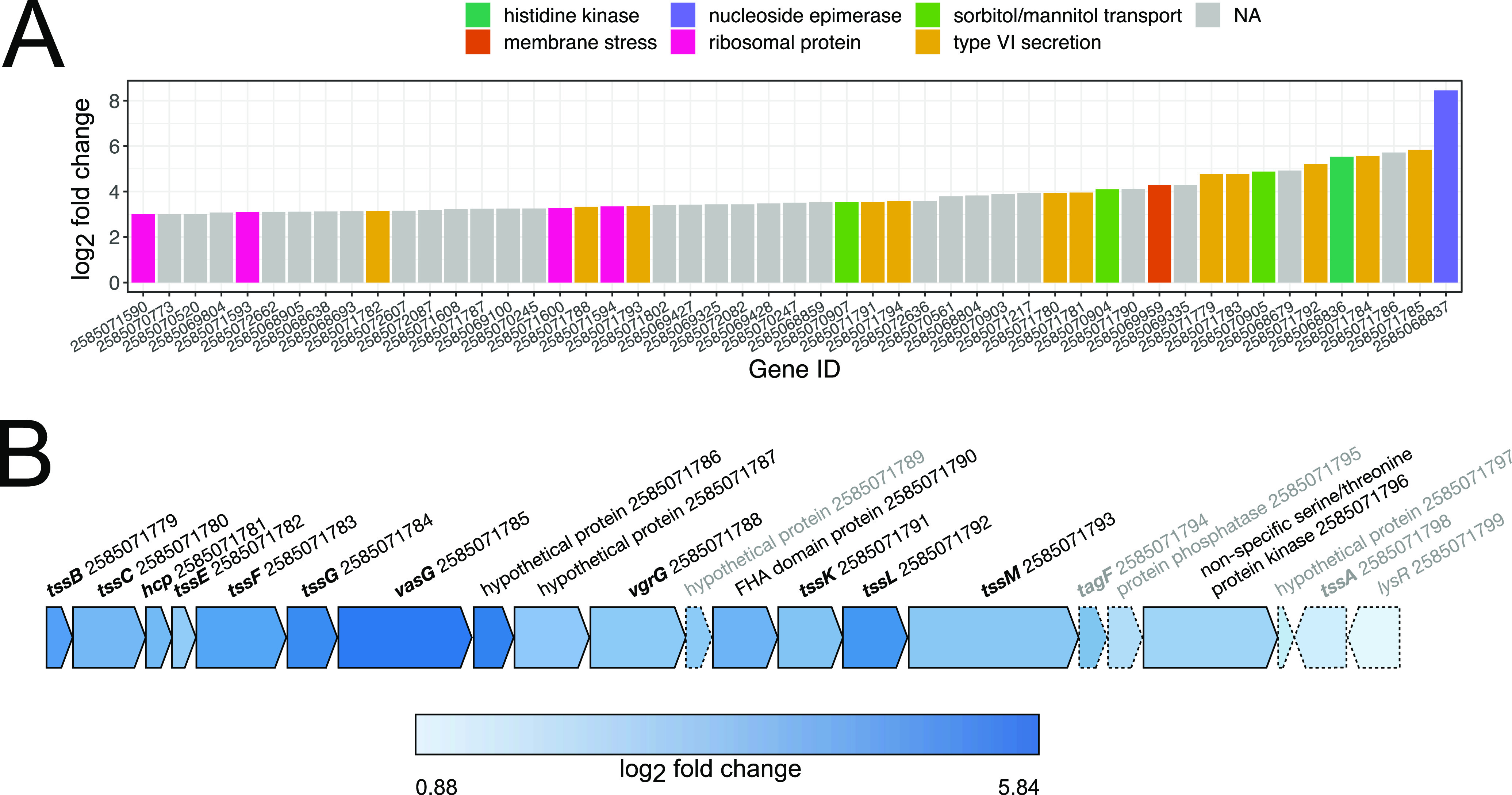
Upregulation of type VI secretion system-related genes in *Luteibacter* sp. strain 9143 when in coculture with *Pestalotiopsis* sp. strain 9143. (A) Log_2_ fold change (L2FC) for significantly highly upregulated genes (i.e., adjusted *P* value of ≤0.01, L2FC of ≥3.0), highlighting important groups of genes, including those associated with the type VI secretion system (T6SS). (B) Physical map showing proximity of genes associated with the T6SS in bold font. Colors indicate L2FC in cocultured versus axenic *Luteibacter* sp. strain 9143. Genes annotated in gray and with hashed lines were neutrally regulated, and all others were upregulated.

Genes more strongly upregulated than the majority of the ribosomal proteins included those involved in the type VI secretion system (T6SS). Most known for antibacterial activity, T6SSs serve various roles, including metal scavenging, biofilm formation, and interactions with eukaryotic predators and hosts (35, 36). In total, 11 of 13 (85%) genes predicted to code for T6SS proteins were upregulated—roughly 24% of the 46 genes observed to be significantly and highly upregulated (i.e., adjusted *P* value of ≤0.01, L2FC of ≥3.0) (Fig. 3; Table S3). Similarly, we detected upregulation of two hypothetical proteins within the cluster that are likely coregulated (Fig. 3; Table S3).

Additionally, the most- and sixth-most-upregulated genes were also clustered, and they were predicted to code for a nucleoside-diphosphate-sugar epimerase and a two-component sensor histidine kinase, respectively (Table S3). Notably, the response regulator in this cluster is also upregulated, although it does not meet our FDR threshold for significance (L2FC = 2.8, *P* = 0.02). Two-component regulatory systems composed of kinases and response regulators are key signal transducers for detection of environmental or cellular changes by bacteria.

We also observed all (4/4) genes predicted to be associated with sorbitol/mannitol transport that form a cluster, as well as one adjacent gene predicted to code for a mannitol dehydrogenase, to be upregulated (Table S3). Similarly, 5 of 15 (33%) genes predicted to code for efflux pumps were upregulated.

### *Luteibacter* downregulates pilus assembly, chemotaxis, and motility in coculture.

We observed many downregulated genes to be associated with type IV pilus assembly, chemotaxis, and motility (Fig. 2). Although all 12 genes predicted to code for type IV secretion system (T4SS) proteins were not DE, 8 of 24 type IV pilus assembly genes were DE, and nearly all (7/8 [88%]) were downregulated (i.e., *pilA*, *pilB*, *pilC*, *pilQ*, *pilV*, and *pilW*). Only one gene, *pilY1*, was upregulated. For the operon consisting of *pilM*, *pilN*, *pilO*, *pilP*, and *pilQ*, only *pilQ* was downregulated.

Whereas several DE genes predicted to be associated with chemotaxis, flagella, and motility are scattered throughout the genome of *Luteibacter* sp. strain 9143, the majority were organized in distinct clusters. For example, we found 18 of 41 genes (44%) predicted to code for flagellar proteins and 8 of 34 genes (24%) predicted to code for chemotaxis proteins to be downregulated. This included genes predicted to code for the sigma-54 specific transcriptional regulator FliA and an anti-sigma-28 factor in the FlgM family, which are two proteins important for regulating the flagellar protein-coding genes (37) (Table S3). Along with 15 of the 18 downregulated genes coding for flagellar proteins and three downregulated genes coding for chemotaxis, these genes form a cluster comprising 47 genes related to chemotaxis and motility (Table S3).

### *Luteibacter* downregulates methionine metabolism in coculture.

When considering amino acid metabolism, we observed genes related to methionine to be DE. For example, all (7/7) genes predicted to code for methionine metabolism or transport were downregulated. This included genes encoding a methionine aminotransferase, a methionine synthase, a methionine adenosyltransferase, two peptide-methionine *S*-oxide reductases (R and S), a methionine transport system substrate-binding protein, and a homoserine *O*-acetyltransferase, the latter of which was the most downregulated gene in the experiment (Table S3). Furthermore, three of four genes involved in leucine biosynthesis and one gene involved in leucine degradation were also downregulated, including those associated with the small and large subunits of 3-isopropylmalate dehydratase, 3-isopropylmalate dehydrogenase, and 2-isopropylmalate synthase and leucine dehydrogenase (Fig. 2). This suggests an undescribed role for leucine or other branched-chain amino acids during the symbiosis.

### Transcriptional changes in *Pestalotiopsis* sp. strain 9143 during coculture may be facilitated by NmrA-like transcription repressors.

**FIG 4:**
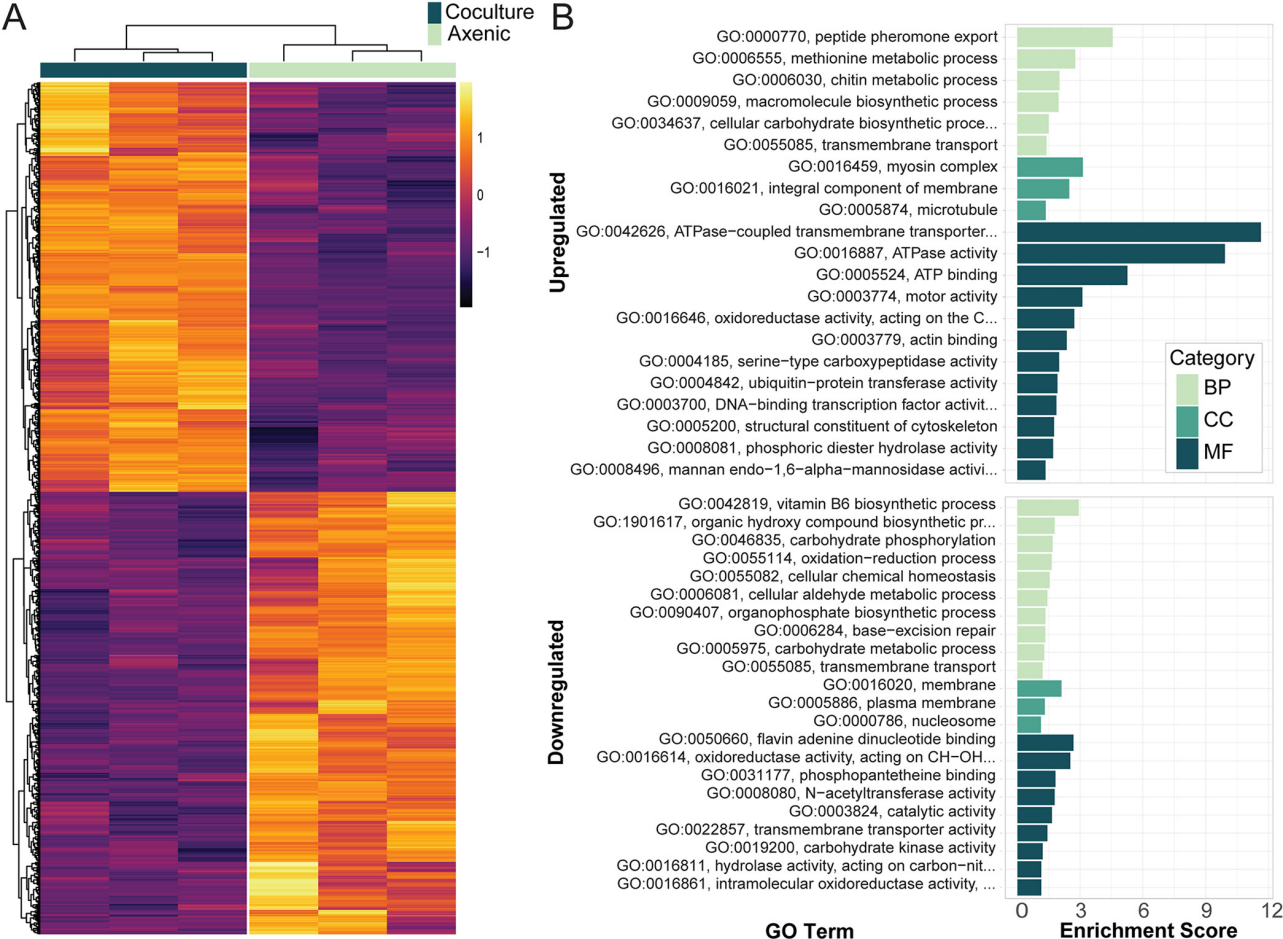
Differentially expressed genes and enriched GO terms in *Pestalotiopsis* sp. strain 9143 when in coculture with *Luteibacter* sp. strain 9143. (A) Heat map of regularized log (rlog)-transformed counts showing the differentially expressed genes identified with DESeq2 for a false-discovery rate of 0.01. Three biological replicates are shown for cocultured and axenic fungal growth. (B) Enriched Gene Ontology (GO) terms among the up- or downregulated differentially expressed genes in cocultured fungus; biological processes (BP), cellular component (CC), and molecular function (MF). Enrichment analysis was done using the topGO R package’s weight01 algorithm and Fisher’s exact test for a *P* value of <0.05.

During coculture with *Luteibacter* sp. strain 9143, *Pestalotiopsis* sp. strain 9143 upregulated 478 genes and downregulated 521 genes, for an FDR of 0.01 (Fig. 4A; see Table S4 in the supplemental material). In contrast to the bacterial genome, the limited number of gene name annotations in the fungal genome made GO analysis a useful tool to identify changes in the fungal transcriptome. There were 21 GO terms enriched in the upregulated genes and 22 GO terms enriched in the downregulated genes (Fig. 4B). The GO terms with the highest enrichment score among upregulated genes were the molecular function (MF) GO terms for ATPase activity (GO:0016887) and ATPase-coupled transmembrane transporter activity (GO:0042626). The top biological processes (BP) GO terms were peptide pheromone export (GO:0000770) and methionine metabolic process (GO:0006555), while the three enriched cellular component (CC) GO terms were myosin complex (GO:0016459), integral component of the membrane (GO:0016021), and microtubule (GO:0005874). The enriched GO terms in the downregulated genes had lower enrichment scores overall, with the largest ones being vitamin B_6_ biosynthetic process (GO:0042819; BP), membrane (GO:0016020; CC), flavin adenine dinucleotide binding (GO:0050660; MF), and oxidoreductase activity, acting on CH-OH group of donors (GO:0016614; MF).

10.1128/msystems.00091-22.6 TABLE S4 Summary of differentially expressed genes in *Pestalotiopsis* sp. strain 9143 when grown in coculture with *Luteibacter* sp. strain 9143. Download Table S4, XLSX file, 2.5 MB. Copyright © 2022 Shaffer et al. 2022 Shaffer et al. https://creativecommons.org/licenses/by/4.0/ This content is distributed under the terms of the Creative Commons Attribution 4.0 International license.

The top two upregulated genes (KJ359_005267 and KJ359_006927) both encode hypothetical proteins with NAD(P)-binding domains that are members of the PFAM NmrA (PF05368), suggesting they are transcription repressors (38). Five additional genes encoding NmrA-like proteins were upregulated, and another one was the ninth-most-downregulated gene. While a few of the most upregulated genes are cytochrome P450 genes, and there are some that are downregulated, these are not enriched in the data set, as only 14 are differentially expressed out of 234 total. Nearly half of the most downregulated genes (i.e., 13 of 30; L2FC ≥ 6) are hypothetical proteins with no annotated domains of known function. The following sections detail the themes present in the differentially expressed genes and GO term enrichment, highlighting the changes in transporter and cell structure-related genes, methionine and carbohydrate metabolism genes, and genes associated with potential defense or signaling compounds like secondary metabolites and β-lactamases.

### Diverse transporters are up- and downregulated by *Pestalotiopsis*.

Transport changes in *Pestalotiopsis* sp. strain 9143 are considerable in the presence of *Luteibacter* sp. strain 9143. The term for transmembrane transport (GO:0055085) is enriched in 42 upregulated genes and 31 downregulated genes that are largely predicted to encode ATP-binding cassette (ABC) transporters or major facilitator superfamily (MFS) transporters, as well as amino acid permeases, oligopeptide transporters, and metal ion transporters. Of the non-MFS and ABC protein-encoding genes, two purine-cytosine permeases (InterPro no. IPR001248) are downregulated. Additionally, three genes putatively encoding magnesium (Mg^2+^) CorA-like transporter proteins are all downregulated, possibly reducing the Mg^2+^ ion concentration and contributing to a reduction in growth and cell wall integrity (39).

Among the downregulated genes associated with transmembrane transport, the majority are genes encoding MFS transporters (21 of 31), and of those, 11 are from the sugar transporter MFS subfamily (IPR005828 and/or PF00083). In contrast, only five predicted sugar transporter genes are upregulated, including the hexose transporter *hxt1*. Among the genes that were upregulated, 19 ABC transporter genes contributed to the most enriched GO terms: ATPase-coupled transmembrane transporter (GO:0042646), ATPase activity (GO:0016887), and ATP binding (GO:0005524). Seven of the 19 are associated with peptide pheromone export (GO:0000770), and only 2 are predicted to be within secondary metabolite clusters: KJ359_000853 in cluster 10.1 is upregulated, and KJ359_002926 in cluster 18.2 is downregulated, as described in detail below. Thus, the substrates of many of these ABC transporters are unknown given the wide variety of potential substrates ranging from lipids to toxins (40).

### Cell structure-related gene expression is impacted by bacterial presence.

Because manipulation of actin is common in close symbioses, such as rhizobial nodule formation in plant roots (41) and intracellular bacterial pathogen motility in animals (42), we predicted that cell wall- and membrane-associated genes would be differentially expressed. We found that the upregulated genes are enriched for many GO terms associated with the cytoskeleton and cell wall: chitin metabolic process (GO:0006030), myosin complex (GO:0016459), microtubule (GO:0005874), motor activity (GO:0003774), actin binding (GO:0003779), structural constituent of cytoskeleton (GO:0005200), and mannan endo-1,6-α-mannosidase activity (GO:0008496). The genes associated with these terms (Table 2) are mostly associated with myosins, chitin synthases, tubulins, and mannan endo-1,6-α-mannosidases. Of the three DE mannan endo-1,6-α-mannosidases, which are typically required for fungal cell growth and contribute to fungal cell wall biosynthesis (43), one is actually downregulated. The DE genes of all other enriched GO terms associated with the cytoskeleton or cell wall are upregulated (Table 2). These results align with the changes in gene expression seen in other bacterial-fungal symbioses; a compatible Rhizopus microsporus isolate upregulates genes involved in cytoskeletal rearrangement and the cell wall when in contact with *Mycetohabitans* spp. (25).

**TABLE 2:** Differentially expressed genes of *Pestalotiopsis* sp. strain 9143 annotated with the enriched GO terms associated with the cytoskeleton and cell wall and their top hit by PSI-BLAST

Gene ID	Annotation	Log_2_ fold change	FDR	Top hit by PSI-BLAST	Organism
KJ359_001200	Hypothetical protein	4.42	1.83E−05	Endochitinase	Manduca sexta
KJ359_008820	Hydrolase 76 protein	2.94	2.54E−04	Mannan endo-1,6-α-mannosidase	Saccharomyces cerevisiae
KJ359_008681	Hypothetical protein	2.68	1.73E−04	Kinesin	Cylindrotheca fusiformis
KJ359_002383	Hypothetical protein	2.62	9.43E−05	Chitotriosidase-1	Homo sapiens
KJ359_012237	Hydrolase 76 protein	1.67	7.40E−04	Mannan endo-1,6-α-mannosidase	Saccharomyces cerevisiae
KJ359_000369	Tubulin β-chain (β-tubulin)	1.38	4.20E−04	Tubulin β-chain	Pestalotiopsis microspora
KJ359_011436	Hypothetical protein	1.32	8.61E−03	Flocculation protein	Saccharomyces cerevisiae
KJ359_012899	Chitin synthase, class 2	1.3	1.78E−03	Chitin synthase 1	Neurospora crassa
KJ359_009573	Hypothetical protein	1.28	8.75E−05	Chitin Synthase 6	Ustilago maydis
KJ359_000339	Hypothetical protein	1.17	2.32E−03	Proline-rich protein	Saccharomyces cerevisiae
KJ359_003752	Chitin synthase, class 1	1.16	5.09E−03	Chitin synthase 3	Neurospora crassa
KJ359_011288	α-Tubulin	1.12	1.65E−03	Tubulin α-B chain	Neurospora crassa
KJ359_012862	Class II myosin	1.11	8.65E−04	Myosin 1	Magnaporthe oryzae
KJ359_010970	Myosin type-2 heavy chain 1	1.11	6.37E−03	Myosin 2	Lachancea kluyveri
KJ359_010970	Myosin type-2 heavy chain 1	1.11	6.37E−03	Myosin 2	Saccharomyces cerevisiae
KJ359_005696	Hypothetical protein	1.06	1.11E−03	Actin-binding protein	Saccharomyces exiguus
KJ359_012986	Hypothetical protein	−3.06	1.93E−13	Mannan endo-1,6-α-mannosidase	Saccharomyces cerevisiae

Notably, many of the genes related to cell structure that were not considered differentially expressed for our set FDR of <0.01 are just beyond that probability threshold and typically have low L2FCs. For example, of the five genes in the *Pestalotiopsis* sp. strain 9143 genome annotated with the term myosin complex (GO:0016459), three genes are DE, with an L2FC of 1.1 to 1.2 for an FDR of <0.01, but the other two genes are DE, for a less-stringent FDR of <0.05. In filamentous fungi, myosins are associated with proper septation within hyphae, sporulation, and cell wall formation (44, 45). Additionally, three of the six genes annotated with the MARVEL domain (IPR008253) are downregulated (KJ359_003932, KJ359_001504, and KJ359_002564), for an FDR of <0.01, whereas two of the remaining three have an FDR of <0.06. Proteins containing the MARVEL domain are not well studied in fungi but have been shown to play a role in actin and membrane organization in Candida albicans (46) and in cell growth and fusion in Neurospora crassa (47).

### Methionine metabolism is upregulated by *Pestalotiopsis*, complementing downregulation by *Luteibacter*.

Within the upregulated genes, the second most significantly enriched GO term for biological processes is that for methionine metabolic process (GO:0006555) (Fig. 4B). Three of 14 genes with this term or child GO terms are slightly upregulated, with an L2FC ranging from 1 to 2: two encode predicted methylenetetrahydrofolate reductases (KJ359_010927 and KJ359_003580) and one a methionine synthase (KJ359_000681). Some proteins involved in methionine metabolism use pyridoxal-5′-phosphate (PLP) as a cofactor. However, two genes encoding PLP synthase subunits Snz1 (KJ359_006612) and PdxT/SNO (KJ359_006611) are downregulated, along with a putative PLP oxidase gene (KJ359_005893). As PLP is a vitamin B_6_ compound, these genes represent the enrichment of the term for vitamin B_6_ biosynthetic process (GO:0042819) among the downregulated genes.

### Glycoside hydrolases account for most changes in *Pestalotiopsis* carbohydrate metabolism during coculture.

We previously observed that cured *Pestalotiopsis* sp. strain 9143 has reduced cellulase activity and reduced growth on cellulose-based medium (3); thus, we expected to see changes in carbohydrate metabolism when cocultured. The majority of the downregulated genes (16 of 23) predicted to be involved in the carbohydrate metabolic process (GO:0005975) encode putative members of glycoside hydrolase families: altogether 15 upregulated and 21 downregulated genes are predicted by InterPro to encode glycoside hydrolases. Glycoside hydrolases act on diverse substrates and are commonly secreted out of the fungal cell to break down biomass, including compounds found in plant cell walls, like lignin, cellulose, and hemicellulose (48). Of the DE glycoside hydrolases, 25 have signal peptides predicted by SignalP (49).

Two downregulated genes involved in the carbohydrate metabolic process (GO:0005975) are also annotated with carbohydrate phosphorylation (GO:0046835) and encode hexokinases. The additional carbohydrate metabolism-associated genes are a glucosamine-6-phosphate isomerase gene, a sugar phosphate isomerase gene (RpiB/LacA/LacB family), and a polysaccharide deacetylase gene. The glucosamine-6-phosphate isomerase (KJ359_003080) converts glucosamine-6-phosphate to fructose-6-phosphate and ammonium in the last step of *N*-acetylglucosamine (GlcNAC) catabolism.

### Only secondary metabolite cluster 10.1 is fully differentially expressed in coculture.

**FIG 5:**
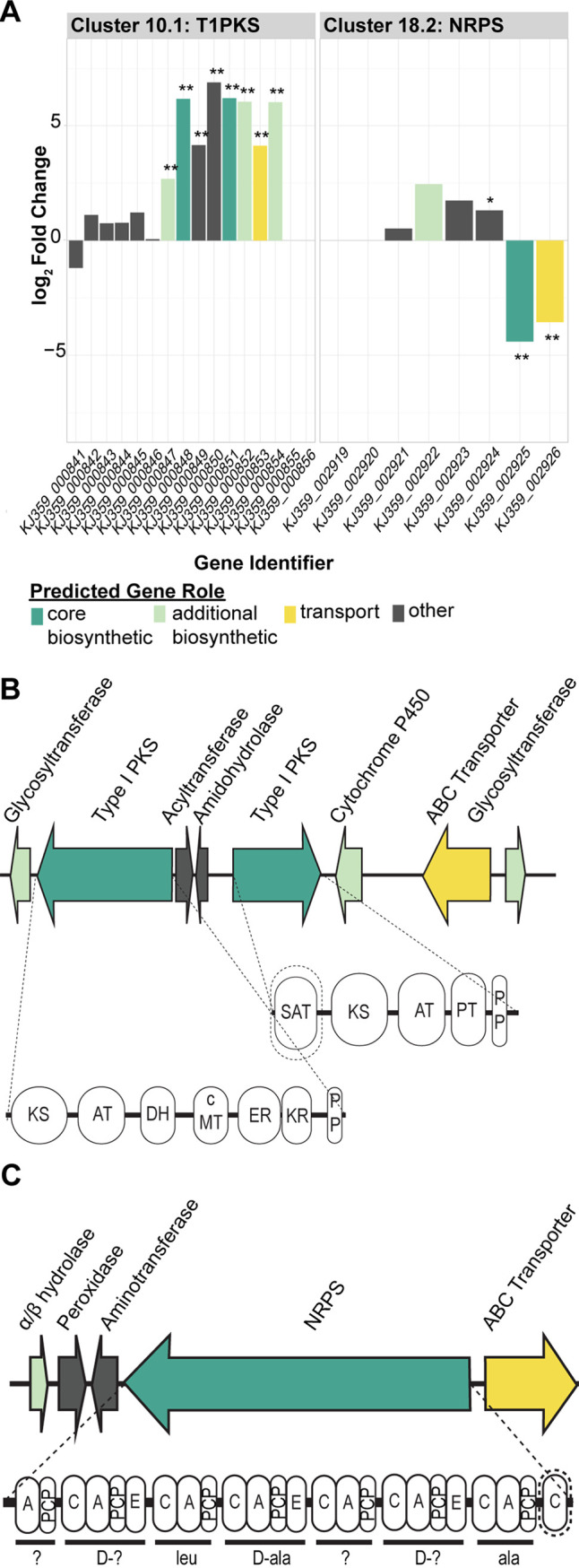
Differential expression of genes within two secondary metabolite clusters of *Pestalotiopsis* sp. strain 9143. (A) Log_2_ fold change of the genes within clusters 10.1 and 18.2 color coded by predicted gene role. Two asterisks (**) designate differentially expressed (DE) genes with an FDR of <0.01; one asterisk (*) designates DE genes with an FDR of <0.05. (B) Diagram of part of the cluster 10.1 type I polyketide synthase (PKS) locus with putative gene products, including PKS domains: ketosynthase (KS), acyltransferase (AT), phosphopantetheine (PP), acyl carrier protein (ACP) dehydratase (DH), enoylreductase (ER), ketoreductase (KR), carbon methyltransferase (cMT), product template (PT), and a N-terminal ACP transacylase similar to the one involved in aflatoxin biosynthesis (SAT). (C) Diagram of part of the cluster 18.2 nonribosomal peptide synthase (NRPS) locus with putative gene products, including NRPS domains: condensation (C), AMP binding (A), epimerization (E), and peptidyl-carrier protein (PCP). Bars underneath groups of ovals indicate NRPS modules and the amino acids they are predicted to load: alanine (ala) and leucine (leu). Incomplete modules or predictions are indicated with a question mark (?).

Of the 64 secondary metabolite clusters (4 of which include neighboring clusters) in the *Pestalotiopsis* sp. strain 9143 genome, 13 have a core biosynthetic gene (i.e., nonribosomal peptide synthase [NRPS], polyketide synthase [PKS], NRPS-PKS hybrid, or terpene cyclase gene) that is DE when cocultured with *Luteibacter* sp. strain 9143 (see Fig. S2 in the supplemental material). Six were downregulated, and five were upregulated. However, only in cluster 10.1 are the core PKS genes and neighboring biosynthetic and transport genes all DE (Fig. 5A and B; Fig. S2). Cluster 18.2 has a DE core NRPS gene (KJ359_002925) and ABC transporter gene (KJ359_002926), but they are downregulated, and the additional α/β-hydrolase gene (KJ359_002922) predicted to be involved in biosynthesis is not DE (Fig. 5A and C; Fig. S2).

10.1128/msystems.00091-22.2 FIG S2 Differential expression of genes within 13 secondary metabolite clusters of *Pestalotiopsis* sp. strain 9143. Shown are the log_2_ fold changes of the genes within secondary metabolite clusters color coded by predicted gene role. Two asterisks (**) designate differentially expressed (DE) genes with an FDR of <0.01; one asterisk (*) designates DE genes with an FDR of <0.05. Download FIG S2, TIF file, 3.3 MB. Copyright © 2022 Shaffer et al. 2022 Shaffer et al. https://creativecommons.org/licenses/by/4.0/ This content is distributed under the terms of the Creative Commons Attribution 4.0 International license.

Cluster 10.1 contains genes putatively encoding two polyketide synthases (PKSs), two glycosyltransferases, a cytochrome P450, and an ABC transporter (Fig. 5B). Additional genes for an acyltransferase and an amidohydrolase were not predicted as serving a role in the cluster, but are upregulated and may also contribute to a final product. The eight genes in total that were upregulated in cluster 10.1 ranged from an L2FC of 2.5 to 7. Both predicted PKS proteins encoded by KJ359_00848 and KJ359_00851 have the required ketosynthase (KS), acyltransferase (AT), and phosphopantetheine (PP) acyl carrier protein (ACP) group domains. KJ359_00848 has a carbon methyltransferase (cMT) domain and three additional reducing domains: dehydratase (DH), enoylreductase (ER), and ketoreductase (KR). In contrast, KJ359_00851 has a product template (PT) domain and an N-terminal ACP transacylase similar to the one involved in aflatoxin biosynthesis (SAT).

### Differentially expressed β-lactamases play an unknown role in fungi.

Five β-lactamase-related (IPR001466) genes were upregulated (1.7 < L2FC < 7.1), while one was downregulated (L2FC = −4.0). In bacteria, β-lactamases confer antibiotic resistance against β-lactams, which are produced by fungi to target bacterial peptidoglycan, with few examples of fungicidal β-lactams (50). Yet, there are few β-lactamase-related genes characterized in fungi, so the computational annotation of β-lactamases in fungal genomes is likely too specific and does not account for the potential diversity of lactamases (51).

## DISCUSSION

We used an RNA-seq experiment to understand the transcriptional responses of the EHB *Luteibacter* sp. strain 9143 and foliar endophyte *Pestalotiopsis* sp. strain 9143 when grown together in coculture versus axenically. Based on previous experiments and preliminary data, we predicted that the bacterium would be mildly parasitic on the fungus and would respond to coculture with its host by altering transcription of genes related to chemotaxis, motility, nutrient acquisition, and secretion systems. Similarly, for the fungus, we predicted changes related to carbohydrates such as cellulose and the upregulation of genes related to repair mechanisms and responses to infection related to symbiosis with *Luteibacter*. These predictions were upheld by our analyses.

In cultures used for the transcriptomic study, we inoculated using a greater ratio of fungus to bacterium than is typically observed in naturally infected fungi (i.e., <10 bacterial cells per fungal hypha), as the low frequency of EHB would make difficult the recovery of bacterial transcripts in the fungal background. In addition to our desire to focus on early stages of the interaction, this is why we used cocultures versus naturally infected *Pestalotiopsis* sp. strain 9143. After the experiment, we observed a greater density of suspended cells of *Luteibacter* sp. strain 9143 in coculture with *Pestalotiopsis* sp. strain 9143 compared to the axenic bacterial culture. This supports previous findings that the bacterium is not able to obtain sulfur in the form of sulfate when grown in pure culture and that growing in coculture with its host fungus can supplement this deficiency (32). Upregulation of genes encoding ribosomal proteins during coculture was consistent with the increased bacterial growth. This finding is even more interesting when considering that the free-living cells of *Luteibacter* sp. strain 9143 that were measured were indeed present among attached and endohyphal cells, implying that the density is even greater for cocultured versus axenic bacteria. At the same time, we observed a greater biomass of *Pestalotiopsis* sp. strain 9143 grown in pure culture compared to that of the fungus growing with its EHB in coculture, implying that fungal growth is inhibited in the presence of *Luteibacter* sp. strain 9143. Whether or not this reduction in growth of the fungus in the presence of the bacterium influences interactions with host plants remains unknown, but should be explored further.

Given the reduction in growth by *Pestalotiopsis* sp. strain 9143 in coculture, the fungus may be undergoing a defense response to *Luteibacter* sp. strain 9143. Indicators of this include the upregulation of diverse transporters and β-lactamase genes, which could be turned on in response to or anticipation of antifungal compounds. For example, a Fusarium verticillioides “metallo-β-lactamase” protein actually confers resistance to plant-derived, antifungal γ-lactams (52). Similarly, the upregulated lactamase genes in *Pestalotiopsis* sp. strain 9143 may play a role in countering antifungal compounds or in improving the environment for putative bacterial partners. Furthermore, it is likely at least a subset of the upregulated transporters mediate resistance to antifungal compounds, especially four differentially expressed genes (KJ359_009703, KJ359_010943, KJ359_010406, and KJ359_003031) that have conserved domains from the pleiotropic drug resistance protein family (PF06422). Antifungal resistance-related gene expression may be in response to *Luteibacter* sp. strain 9143 specifically or bacteria generally, and these may be fruitful avenues to explore for host specificity in bacterial-fungal interactions.

The responses of *Luteibacter* and *Pestalotiopsis* to one another in coculture include bacterial cells in multiple stages of interaction, including free-living, attached, entering, and endohyphal, complicating interpretation of the transcriptional response. This may contribute to why we see low L2FCs for cell wall- and membrane-associated genes in the fungus, as the majority of bacterial cells are likely external to the fungus. Still, we see the response of *Luteibacter* sp. strain 9143 in coculture with *Pestalotiopsis* sp. strain 9143 as representing an immediate metabolic response to changes in environmental conditions and the initiation of association with the fungal partner. This includes the downregulation of methionine metabolism, chemotaxis, and motility and the upregulation of sorbitol and mannitol transport and the T6SS (Fig. 2 and 3). In particular, we speculate that the upregulation of the T6SS may help *Luteibacter* to initiate and establish symbiosis with *Pestalotiopsis*, although we recognize that the bacterium may be using the T6SS for other purposes, such as nutrient acquisition or even defense (36, 53, 54). Whereas it is difficult to understand which metabolites may be exchanged during this interaction from these genomic data, future studies using metabolomics may provide additional insight (e.g., reference 30).

Metabolic studies also could illuminate the role secondary metabolites play in facilitating the establishment of fungal-bacterial partnerships, including both recruitment and invasion. In the R. microsporus and *Mycetohabitans* interaction, secondary metabolites are not thought to play a large role, as only one NRPS is upregulated and one PKS downregulated in R. microsporus (i.e., the natural host strain) when in contact with the bacterium (25). In contrast, the Ralstonia solanacearum lipopeptide ralsolamycin induced substantial developmental shifts in host fungi, enhancing bacterial entry into fungal chlamydospores (30). In our analysis, *Pestalotiopsis* differentially expresses the core biosynthetic gene in 13 secondary metabolite clusters, but only cluster 10.1 had all predicted biosynthetic genes differentially expressed. Given the two PKS genes in cluster 10.1, it is possible that two separate metabolites are made, but the coregulation and tight clustering support a single metabolic pathway. For example, a single Aspergillus nidulans cluster creates asperfuranone using two PKS genes—one highly reducing and the other not—along with five additional genes (55). Cluster 10.1 does not appear to be related to the asperfuranone cluster based on the difference in accessory gene content and lack of homology between KJ359_00848 and KJ359_00851 and the asperfuranone PKS genes, though it does similarly contain one PKS gene predicted to be highly reducing and one not. Notably, this cluster is present in Neopestalotiopsis clavispora, *Neopestalotiopsis* sp. strain 37M, and Pestalotiopsis microspora at nearly 80% protein identity, suggesting conservation in closely related fungi. We also observed *Luteibacter* sp. strain 9143 to upregulate genes predicted to code for multidrug efflux pumps, which may be in response to the production of secondary metabolites by *Pestalotiopsis* sp. strain 9143 and assist the bacterium in colonizing its host, especially if the fungus is producing them as a defense response to bacterial invasion.

Our study supports a more direct exchange of primary metabolites, as *Luteibacter* sp. strain 9143 cannot utilize the sulfate in the minimal medium as a sulfur source, thus requiring methionine or other organic sulfur compounds generated by the fungus (32). Methionine is limited in the plant apoplast, and methionine synthases are critical in plant-pathogenic ascomycetes for survival *in planta* (56, 57). The small upregulation of methionine synthesis genes by the fungus may indicate a feedback response to having sulfur-containing compounds be depleted by the presence of *Luteibacter* sp. strain 9143, or it may be more actively encouraged by the bacterium in some way. In Saccharomyces cerevisiae, excess methionine is tied to upregulation of synthesis of the cofactor pyridoxal-5′-phosphate (PLP) (58), as part of an anabolic program leading to increased synthesis of amino acids. The downregulation of PLP synthases in *Pestalotiopsis* sp. strain 9143 could be tied to decreased methionine availability based on bacterial use, potentially leading to less amino acid metabolism and contributing to the observed growth restriction. Interestingly, genes predicted to be associated with methionine metabolism were downregulated in *Luteibacter* sp. strain 9143 (Fig. 2B), which also supports the hypothesis that the bacterium is acquiring methionine from *Pestalotiopsis* sp. strain 9143.

Altogether, our transcriptomic analysis supports the phenotypic findings we have observed in the *Pestalotiopsis-Luteibacter* partnership, including a dependence on the fungal host as a sulfur source for *Luteibacter*, a change in extracellular carbohydrate metabolism by *Pestalotiopsis* when *Luteibacter* is present, and inverse growth impacts on the partners during coculture. Together, these findings support a working model in which *Luteibacter* switches from a motile to sedentary lifestyle in association with the fungus, which can provide it with methionine as a sulfur source. Other metabolites, such as leucine, mannitol, or sorbitol, may also be exchanged. The specific metabolites and signaling pathways leading to the transcriptional changes remain to be investigated, but may involve the highly upregulated bacterial two-component system or T6SS and the fungal PKS secondary metabolite cluster 10.1 or NmrA repressors. Future work should attempt to track responses of *Luteibacter* sp. strain 9143 as it attaches and becomes endohyphal with *Pestalotiopsis* sp. strain 9143, in addition to exploring later stages of the interaction. Based on the diversity of EHB-fungal relationships, additional work to probe the mechanisms underlying this and other partnerships will be critical for informing a broader model of bacterial-fungal interactions and, especially, how they relate to ecology in the phyllosphere.

## MATERIALS AND METHODS

We obtained *Pestalotiopsis* sp. strain 9143 from a living culture collection at the Robert L. Gilbertson Mycological Herbarium, University of Arizona, Tucson. The fungus was isolated originally as an endophyte from healthy, asymptomatic foliage of Platycladus orientalis (Cupressaceae) (59). *Pestalotiopsis* sp. strain 9143 was naturally infected with the endohyphal bacterium *Luteibacter* sp. strain 9143 at the time of isolation and maintained a consistent infection throughout growth in culture on various media and vouchering in sterile water. The bacterium was isolated successfully in culture, and a rifampin-resistant strain (9143) was generated by plating on lysogeny broth (LB) amended with 50 μg/mL rifampin (31).

To generate cured *Pestalotiopsis* sp. strain 9143, conidia from sporulating, 21-day-old cultures were transferred to a new petri plate (60-mm) containing 2% malt extract agar malt extract agar (MEA) amended with four antibiotics: tetracycline (10 μg/mL), ampicillin (100 μg/mL), ciprofloxacin (40 μg/mL), and kanamycin (50 μg/mL) (MEA+TACK). Total genomic DNA extracted from fungal mycelium was used for PCR to screen for the presence or absence of *Luteibacter* sp. strain 9143 by amplifying the bacterial 16*S* rRNA (rRNA) gene as described in reference 15. Successful amplification was assessed by mixing PCR products with SYBR green and running on a 2% agarose gel in Tris-EDTA buffer (1×).

### Culture conditions prior to RNA extraction.

After confirming the absence of *Luteibacter* sp. strain 9143 in *Pestalotiopsis* sp. strain 9143 growing on MEA+TACK, conidia were transferred to a new MEA plate. We then revived *Luteibacter* sp. strain 9143 by streaking from glycerol stock onto LB agar (1% NaCl [i.e., LB-Miller]). Both fungal and bacterial cultures were incubated under ambient laboratory conditions. After 21 days, for *Pestalotiopsis* sp. strain 9143, six ca. 2-mm^2^ squares were excised from the growing edge of the fungal colony with a sterile toothpick and transferred to a sterile 125-mL flask containing 50 mL fresh liquid M9 plus glucose (2%) plus methionine (10 mM) (high-methionine minimal) medium. In parallel for *Luteibacter* sp. strain 9143, a sterile toothpick was used to transfer bacterial cells from a single colony to a test tube containing 5 mL fresh liquid high-methionine minimal medium. Fungal and bacterial cultures were then incubated at 27°C with shaking at 200 rpm.

After 10 days, we processed cultures to produce one culture flask for each of the following treatments: (i) *Pestalotiopsis* sp. strain 9143 grown alone (i.e., axenic fungus), (ii) *Luteibacter* sp. strain 9143 grown alone (i.e., axenic bacterium), and (iii) *Pestalotiopsis* sp. strain 9143 and *Luteibacter* sp. strain 9143 grown together (i.e., fungus plus bacterium coculture). The fungal inoculum was created by transferring mycelia from the fungal culture flask to a sterile 50-mL stainless steel Sorvall Omni mixer homogenizer chamber assembly (5.08-cm blade, polytetrafluoroethylene [PTFE] bearings; Omni International, Inc., Kennesaw, GA, USA) containing 15 mL fresh liquid high-methionine minimal medium for homogenization: 20 s on, 1 min off, and 20 s on. The bacterial inoculum was created by diluting the liquid culture to an optical density at 600 nm (OD_600_) of 0.1. Five milliliters of mycelial suspension and of bacterial suspension (i.e., ∼4.0 × 10^8^ bacterial cells) was added to flasks with fresh liquid high-methionine minimal medium to create an axenic fungal culture, an axenic bacterial culture, and a fungal-bacterial coculture, each at a final volume of 50 mL. Whereas the frequency of bacterial cells among naturally infected fungal hyphae can be highly variable, we used this density of bacteria to better capture both partners’ response during the early stages of interaction.

After 4 days, we split cultures to produce three biological replicates for each of the three treatments above. We first transferred the contents of each culture flask to a sterile 50-mL Falcon tube (Corning, NY, USA), centrifuged at 11,000 rpm for 20 min (5430R; Eppendorf, Hamburg, Germany) to pellet cells, removed the medium by pipetting, and resuspended the cells in fresh liquid M9 plus glucose (2%) plus methionine (100 μM) (low-methionine minimal) medium to bring the total volume of each tube to 25 mL. We then transferred 5 mL of each culture to four sterile 125-mL flasks containing 45 mL fresh liquid low-methionine minimal medium and incubated all cultures at 27°C with shaking at 200 rpm. Previous work indicates *Luteibacter* sp. strain 9143 cannot utilize sulfate as a sulfur source during laboratory growth, but is able to grow in medium supplemented with cysteine, methionine, or high levels of thiosulfate, as well as in coculture with its host fungus, *Pestalotiopsis* sp. strain 9143 (32). Although we used a high-methionine medium in order to allow for robust growth of *Luteibacter* sp. strain 9143 up to this point, we now reduced its concentration in hopes of inducing association of *Luteibacter* sp. strain 9143with *Pestalotiopsis* sp. strain 9143 in coculture, as the bacterium appears to acquire sulfur from the host fungus (32).

### Bacterial density and total biomass of experimental cultures.

After 3 days and immediately prior to RNA extraction, we quantified the density of free-living *Luteibacter* sp. strain 9143 (i.e., those cells not associated with fungal mycelium in coculture) in all cultures by removing 50 μL of liquid culture, diluting 1:1 M, spreading 50 μL onto the surface of a petri plate (100-mm) containing LB agar, and incubating at 27°C for 48 h prior to counting CFU. Following plating, we recovered and lyophilized all remaining tissue in each culture by first transferring to a 50-mL Falcon tube and centrifuging at 11,000 rpm for 20 min to pellet cells. We avoided manipulating the cocultured flasks such as to isolate only bacteria associated with fungal mycelia, in order to prevent transcriptional responses (e.g., such as to washing). Importantly, we note that the population of *Luteibacter* sp. strain 9143 in cocultured flasks includes free-living, externally associated, and endohyphal cells. We next transferred each pellet to a distinct, sterile 1.7-mL microcentrifuge tube with the lid punctured using a sterile needle and immediately submerged samples in liquid nitrogen until the boiling stopped. We then transferred sample tubes to a precooled lyophilizer flask and lyophilized samples for 24 h prior to storing them at −80°C.

### Extraction of RNA from experimental cultures.

For axenic fungal and cocultured samples, we transferred lyophilized tissue to a preweighed, sterile 1.7-mL microcentrifuge tube, ground tissue using a sterile pestle, and obtained total biomass by weighing and subtracting the weight of the tube. For axenic bacterial samples, we transferred 1.5 mL suspended cells to a sterile 1.7-mL microcentrifuge tube, pelleted cells, removed excess medium, and flash froze before lyophilizing cells. To extract RNA, for each sample we used either 0.05 g tissue (ca. 100 μL of ground tissue; axenic fungal and cocultured samples) or entire lyophilized cell pellets (axenic bacterial samples). For each sample, biomass was added to a sterile 1.7-mL microcentrifuge tube containing sterile zirconium oxide beads. We then added 1 mL TRIzol to each sample tube, macerated samples using a bead beater run for 2 min (power level 10), incubated samples on ice for 20 min, and centrifuged at 12,000 rpm for 15 min at 4°C (5804 Eppendorf) to pellet tissue. We next transferred supernatants of each sample to sterile 1.7-mL microcentrifuge tube, added 250 μL chloroform, and mixed all samples uniformly by placing them in a single tube rack, shaking the rack for 15 s, allowing it to rest for 15 s, and shaking again for 15 s. We then incubated samples under ambient laboratory conditions for 5 min, centrifuged at maximum speed for 15 min at 4°C (5804 Eppendorf) to separate phases, transferred 400 μL of the upper, aqueous layer of each sample to a distinct, sterile 1.7-mL microcentrifuge tube, discarded the pellet, and added 750 μL of prechilled (–20°C) ethanol (100%) to each sample tube. We inverted sample tubes several times uniformly as described above, and incubated them on ice for 10 min. Nucleotides were observed precipitating in each sample. We next centrifuged sample tubes at maximum speed for 15 min at 4°C as described above, removed and discarded supernatant from each sample tube by pipetting, washed each pellet with 1 mL ethanol (75%), decanted the supernatant, and added 1 mL ethanol (75% with diethyl pyrocarbonate [DEPC] water) to each sample tube. We then centrifuged samples at 14,000 rpm for 5 min at 4°C, decanted the supernatant, and dried the pellets by centrifugation in an Eppendorf Vacufuge at 12,000 rpm for 3 min at 30°C. We next resuspended each pellet in 40 μL DEPC water and incubated sample tubes for 5 min at 65°C, prior to storage at −80°C.

We quantified the 260/280 (i.e., nucleotide/protein) and 260/230 (nucleotide/organic) ratios for each sample using a NanoDrop (pre-DNase ratios), prior to treatment with DNase using the DNA-free kit (Ambion AM1906; Thermo Fisher, Waltham, MA, USA). Briefly, for each sample we transferred a volume equivalent to 20 μg total nucleotide to a sterile 1.7-mL microcentrifuge tube and added DEPC water to obtain a total volume of 45 μL. We then added DNase I buffer to each sample, vortexed gently, added 1 μL rDNase, vortexed gently, and incubated sample tubes for 30 min at 35°C. For all samples, we then repeated adding 1 μL rDNase, vortexing, and incubating for 30 min at 35°C. We next added 5 μL DNase I inactivation suspension to each sample tube and incubated under ambient laboratory conditions for 2 min, hand-vortexing every 30 s. We quantified 260/280 and 260/230 ratios for each sample again as described above (post-DNase ratios), diluted each sample to a concentration of 400 ng/μL in DEPC water to produce a total volume of 50 μL, and submitted sample tubes to the University of Arizona Genetics Core (UAGC) for quality checking, rRNA depletion and poly(A) selection, and sequencing.

### RNA sequencing.

From all fungal RNA samples, fungal rRNA was depleted twice with the Ribominus transcriptome isolation kit for yeast (Thermo Fisher), and poly(A) selection to acquire fungal transcripts was done using the NEBNext poly(A) mRNA magnetic isolation module (New England BioLabs). The remaining RNA from the cocultured samples and the RNA from the axenic bacterial samples were depleted twice of bacterial rRNA using the Ribominus transcriptome isolation kit for bacteria (Thermo Fisher). All subsequent quality checking was performed by the University of Arizona Genomics Core (UAGC) using an Agilent Bioanalyzer 2100 and RNA 6000 Pico Chips. Sequencing libraries were prepared using RNA TruSeq library construction kits (Illumina, San Diego, CA, USA) and sequenced on an Illumina HiSeq 2500 using TruSeq 2 × 100-bp paired-end chemistry (Illumina) by UAGC.

### Sequencing and assembly of *Pestalotiopsis* sp. strain 9143 genome.

Genomic DNA of *Pestalotiopsis* sp. strain 9143 was prepared as described in reference 60. Genomic DNA was sent to the Microbial Genome Sequencing Center (MiGS) for library preparation using an Illumina tagmentation kit and paired-end (2 × 150 bp) sequencing with a NextSeq 550 instrument as per reference 61. Long-read sequencing was done in the Baltrus lab from the same genomic DNA sample. DNA was prepared for sequencing using the LSK109 ligation sequencing kit without shearing and using the “Long Fragment” buffer. Reads were sequenced using an R9.4 flow cell in MinION, with base calling by Guppy (v3.2.6).

Illumina and Nanopore sequencing reads were used to create a *de novo* hybrid assembly with MaSuRCA (v3.4.1) (62, 63). The resulting assembled genome (see Table S5 in the supplemental material) FASTA file was used as input for gene prediction and annotation by the funannotate (v1.7.4) pipeline (64). Repetitive contigs were removed, and the remaining contigs were sorted before repeat masking. RNA-seq reads assembled with Trinity (65) were used as transcript evidence for gene prediction of the softmasked genome. Putative functional annotation by funannotate (see Table S6 in the supplemental material) was informed by searches of UniProt DB version 2020_04, antiSMASH 5.0 (34), Phobius (66), InterproScan5 (67), dbCAN v9.0 (68), MEROPS v12.0 (69), and eggNOG-mapper v1.0.3 (70). Two scaffolds were flagged as part of the mitochondrial genome and annotated with MITOS (71). The genome assembly and annotations were deposited in NCBI Genomes, and the raw reads were deposited in the Sequence Read Archive.

10.1128/msystems.00091-22.7 TABLE S5 Assembly statistics for the genome of *Pestalotiopsis* sp. strain 9143 from MaSuRCA. Download Table S5, XLSX file, 0.01 MB. Copyright © 2022 Shaffer et al. 2022 Shaffer et al. https://creativecommons.org/licenses/by/4.0/ This content is distributed under the terms of the Creative Commons Attribution 4.0 International license.

10.1128/msystems.00091-22.8 TABLE S6 Gene annotation frequency across various tools within the funannotate pipeline. Download Table S6, XLSX file, 0.01 MB. Copyright © 2022 Shaffer et al. 2022 Shaffer et al. https://creativecommons.org/licenses/by/4.0/ This content is distributed under the terms of the Creative Commons Attribution 4.0 International license.

### Analysis of RNA-seq.

Illumina data were quality controlled using QoRTs (72). We mapped fungal reads onto the genome of *Pestalotiopsis* sp. strain 9143 (JAHZSN000000000.1) and bacterial reads onto that of *Luteibacter* sp. strain 9143 (JQNL00000000.1) (18) by using STAR v2.7.3 (73). Mapped reads were subset with SAMtools (74), and transcript abundances were quantified using featureCount, part of the subread (v2.0.1) package (75) (see Tables S7 and S8 in the supplemental material). For *Luteibacter* sp. strain 9143, we observed a higher proportion of unmapped reads from coculture samples and found that the majority of those reads (i.e., between 70 and 75%) aligned to the genome of *Pestalotiopsis* sp. strain 9143. Whereas this confirms our expectation that our upstream rRNA depletion and poly(A) selection were not 100% efficient (Table S7), the number of aligned reads is sufficient and informative. One axenic fungal sample had triple the reads from sequencing, so the reads were randomly downsampled with SAMtools to be more closely aligned with the number of reads from the other samples (Table S8). We carried out analysis of differential gene expression using the R package DESeq2 (76, 77), with a false-discovery rate (FDR) value of 0.01 as a threshold for differential expression. We looked for enrichment for specific Gene Ontology (GO) terms among all differentially expressed genes for both *Pestalotiopsis* sp. strain 9143 and *Luteibacter* sp. strain 9143 (i.e., grown in coculture versus axenically). Separately for the fungus and the bacterium, we first generated a list of differentially expressed genes and corresponding GO annotations based on the reference genome and then conducted a functional enrichment analysis with a Fisher’s exact test with a *P* value of 0.01 (78), using the R package topGO (79).

10.1128/msystems.00091-22.9 TABLE S7 RNA-seq read mapping and feature count results for *Luteibacter* sp. strain 9143. Shown are read counts of RNA from cocultures after depletion of fungal mRNA as well as bacterial and fungal rRNA. We note that the higher proportion of unmapped reads in cocultures versus axenic reflects fungal RNAs remaining following this procedure, which was expected. Download Table S7, XLSX file, 0.01 MB. Copyright © 2022 Shaffer et al. 2022 Shaffer et al. https://creativecommons.org/licenses/by/4.0/ This content is distributed under the terms of the Creative Commons Attribution 4.0 International license.

10.1128/msystems.00091-22.10 TABLE S8 RNA-seq read mapping and feature count results for *Pestalotiopsis* sp. strain 9143. Shown are read counts for RNA from cocultures depleted of fungal rRNA and enriched for fungal mRNA. Download Table S8, XLSX file, 0.01 MB. Copyright © 2022 Shaffer et al. 2022 Shaffer et al. https://creativecommons.org/licenses/by/4.0/ This content is distributed under the terms of the Creative Commons Attribution 4.0 International license.

Finally, we recognize that independent validation of RNA-seq results is a long-standing and important aspect of gene expression studies. However, in line with current community expectations (80), our conservative stringency thresholds and consistency between biological replicates do not indicate that independent validation of expression would meaningfully improve interpretation of our experimental data.

### Data availability.

The genome assembly and annotations were deposited in NCBI Genomes (accession no. JAHZSN000000000.1), and the raw reads were deposited in the Sequence Read Archive (accession no. SRR14629398 and SRR14629399). All sequencing from this study can be accessed through BioProject no. PRJNA732082 and PRJNA750803. Raw sequencing reads and processed featureCounts tables can be accessed through the NCBI Gene Expression Omnibus (accession no. GSE181155).
