# Supplementary figures and images for: Transcriptional Profiles of a Foliar Fungal Endophyte (Pestalotiopsis, Ascomycota) and Its Bacterial Symbiont (Luteibacter, Gammaproteobacteria) Reveal Sulfur Exchange and Growth Regulation during Early Phases of Symbiotic Interaction
Source: mSystems. 2022 Mar 16;7(2):e00091-22. doi: 10.1128/msystems.00091-22 (PMC9040847; doi:10.1128/msystems.00091-22)

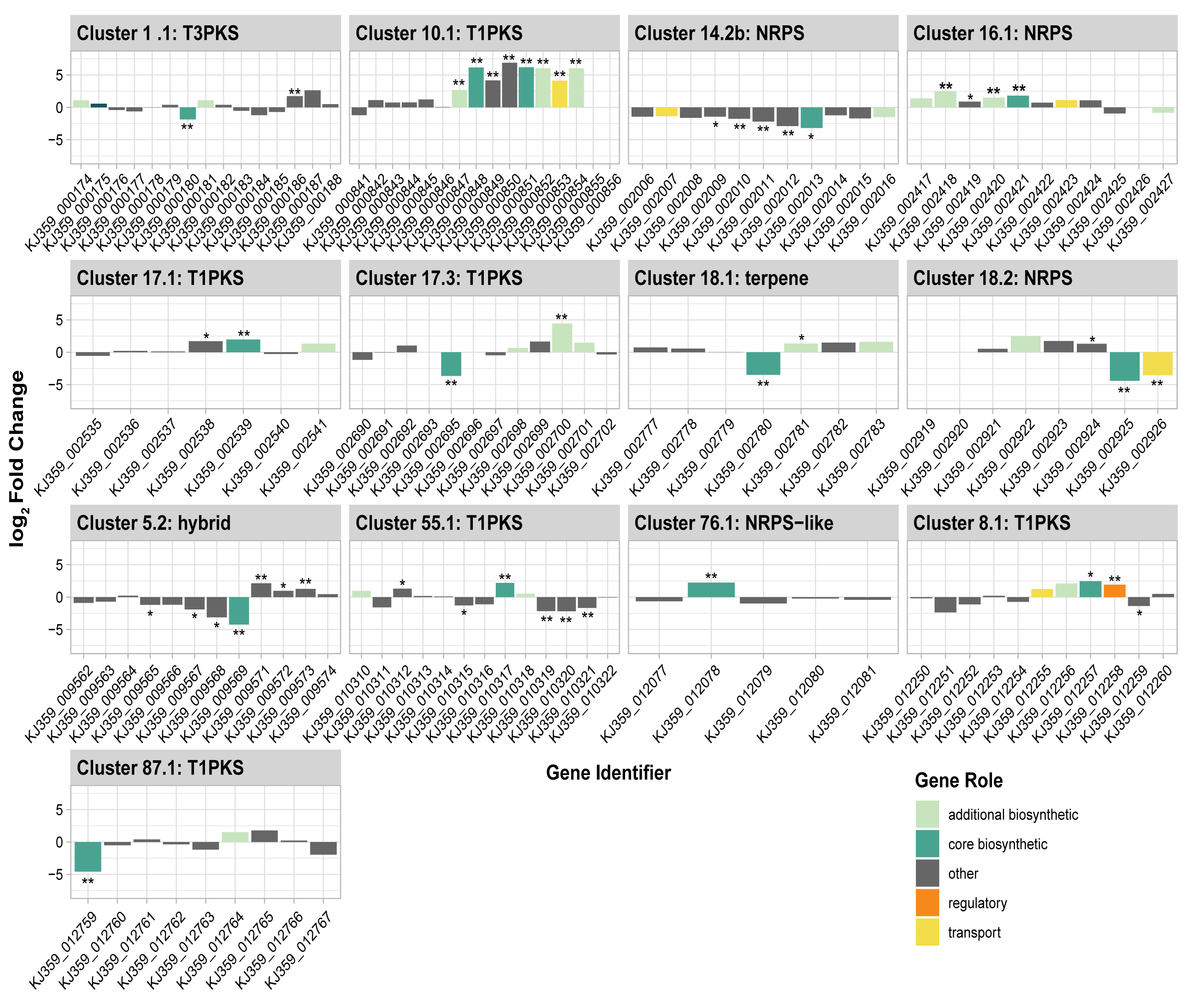

Supplement: FIG S2 [file msystems.00091-22-sf002.tif]
